# Postbiotic supplementation stabilizes gingivitis and improves zootechnical performance of Brangus heifers in an extensive grazing system

**DOI:** 10.3389/fvets.2025.1720339

**Published:** 2026-01-16

**Authors:** Júlia Rebecca Saraiva, Jonatas Campos de Almeida, Ricardo Pereira Manzano, Sabrina Pereira Bomfim Beda, Marcello Pasquale Riggio, Iveraldo Santos Dutra, Ana Carolina Borsanelli

**Affiliations:** 1 Department of Veterinary Medicine, School of Veterinary Medicine and Animal Science, Universidade Federal de Goiás, Goiânia, Brazil; 2 Veterinary Consultant at NUTRIPEC, Catanduva, Brazil; 3 Dental School, University of Glasgow, Glasgow, United Kingdom; 4 Department of Production and Animal Health, Araçatuba School of Veterinary Medicine, São Paulo State University (UNESP), Araçatuba, Brazil

**Keywords:** beef cattle, gingivitis, postbiotic, protein-energy supplement, *Saccharomyces cerevisiae*

## Abstract

Gingivitis represents an early and reversible stage of periodontal disease in cattle and may influence animal welfare and productivity. Controlling this condition in grazing systems remains challenging, and nutritional strategies could represent a sustainable approach to oral health management. This study evaluated the effect of three dietary supplements on the occurrence of gingivitis in incisor teeth and on weight performance of 150 crossbred Brangus beef heifers maintained on *Brachiaria* pasture for a period of 4 months. The animals were allocated into three groups (*n* = 50 per group): (1) protein–energy supplement with *S. cerevisiae* postbiotic, (2) standard protein–energy supplement, and (3) mineral supplement (control). Gingival health was assessed monthly by oral examination of deciduous incisor teeth, and body weight was recorded to determine average daily gain (ADG). No significant differences in gingivitis frequency were observed among groups (*p* > 0.05). However, intra-group analysis showed that only the postbiotic-supplemented group maintained stable gingivitis frequency throughout the trial, while the other groups exhibited significant increases (*p* < 0.001). The postbiotic-supplemented heifers achieved higher ADG (0.300 kg/day) than those receiving the standard protein supplement (0.258 kg/day) and mineral supplement (0.117 kg/day) (*p* = 0.011). Postbiotic supplementation with *S. cerevisiae* did not reduce gingivitis occurrence but helped prevent its progression and improved weight performance. These findings indicate that postbiotics may contribute to maintaining oral health and productivity in grazing cattle, representing a promising nutritional strategy for sustainable livestock systems.

## 1 Introduction

Periodontal diseases, including gingivitis and periodontitis, are multifactorial conditions with variable prevalence and manifestations in cattle herds (1–4). Their occurrence in ruminants is associated with factors such as diet (5, 6), soil management (7), and age (8). Gingivitis represents the earliest and reversible stage of these disorders and is therefore a key target for preventive interventions (9). Despite its relevance, gingival inflammation in grazing cattle remains largely overlooked in veterinary research, and effective control strategies are limited.

Controlling oral diseases in ruminants remains a challenge in animal production. Traditional oral hygiene practices are unfeasible in livestock, highlighting the need for alternative approaches capable of modulating local inflammatory responses through systemic or nutritional routes (9). In ruminant nutritional management, the strategic use of feed additives enhances animal productivity by modulating the ruminal microbiome and fermentation processes (10) and also represents a sustainable alternative to conventional antibiotics (11). Among the main categories of additives used for this purpose are probiotics, prebiotics, and postbiotics, which not only improve zootechnical performance but also support the growth of the livestock feed sector.

Postbiotics are bioactive molecules prepared from a combination of microbial cells or inactivated cellular components and metabolic by-products. They act directly by modulating immune responses and gene expression, exhibiting antimicrobial, antioxidant, and immunomodulatory properties (12). Probiotics, in turn, are live microorganisms that confer benefits when administered in functional doses (13), whereas prebiotics are components that selectively stimulate the growth of beneficial microorganisms in the gastrointestinal tract (14).

In the oral cavity, probiotics can act by preventing the formation of biofilms, reducing salivary pH, and producing antioxidants. Among the most important components of postbiotics, organic acids are recognized for their different antimicrobial mechanisms. In addition to their biological benefits, postbiotics are clinically safe and can be easily incorporated into different food formulations (15). In livestock production systems, postbiotics promote additional physiological and metabolic benefits to animal health, particularly through the modulation of immune responses, inflammatory processes, and the balance of the gastrointestinal tract (16, 17).

In this context, nutritional yeasts deserve special attention due to their rich composition in proteins, essential amino acids, energy compounds, and micronutrients (16). In cattle, the use of yeast-based probiotics and postbiotics derived from *Saccharomyces cerevisiae* has shown benefits for immunity, productive performance, and ruminal microbiome modulation (18–20). In high-producing dairy cows, supplementation with *S. cerevisiae* has led to increased milk yield and fat content, higher ruminal acetate production, and reduced inflammatory cytokine levels (20). In feedlot cattle, *S. cerevisiae* culture supplementation has been associated with increased average daily gain, improved feed conversion, higher digestibility, and consequently, better carcass gains (21). However, their potential effects on oral health in ruminants have not been previously investigated.

Given the increasing emphasis on sustainable nutrition and antibiotic alternatives in animal production, understanding whether postbiotic supplementation can influence oral health represents a novel and relevant research avenue. Therefore, this study aimed to evaluate the effects of *S. cerevisiae*-derived postbiotics on gingival health and zootechnical performance in grazing Brangus heifers. We hypothesized that postbiotic supplementation could stabilize gingivitis occurrence and enhance weight gain under extensive pasture conditions.

## 2 Materials and methods

### 2.1 Ethics statement

The experiment was approved by the Animal Use Ethics Committee (CEUA) of the Federal University of Goiás (UFG) (Protocol number 096/24).

### 2.2 Experimental design and study population

The experiment was conducted on a farm located in the municipality of Paulistânea, in the State of São Paulo, Brazil (Latitude: 22°32′21.9” S, Longitude: 49°11′38.2” W). A total of 150 Brangus beef heifers and crossbreeds, aged between 12 and 14 months at the beginning of the study, were monitored. The animals were randomly assigned into three groups with homogeneous initial weights, according to the supplementation used in the experimental design: (1) protein-energy supplement with a yeast-derived postbiotic (*S. cerevisiae*) (*n* = 50); (2) standard protein-energy supplement (*n* = 50); and (3) mineral supplement (*n* = 50). The experiment lasted 4 months, including a 20-day adaptation period. The heifers were weighed monthly and subjected to clinical oral examinations based on the criteria described by Borsanelli et al. (1). All animals received the recommended clinical and sanitary care throughout the entire experimental period, including regular parasite control. No intercurrent events were recorded during the conduct of the study.

### 2.3 Diet composition

The experiment was conducted under continuous grazing on *Brachiaria decumbens* pasture, in an area ranging from 10 to 20 hectares, without any agricultural management such as pasture renewal or soil treatment in the past 30 years. Forage availability was visually monitored and adjusted throughout the experimental period. The study was conducted during the dry season, and no significant climatic variations were observed. The chemical and bromatological composition and estimated total nutrients of the protein-energy supplement were as follows: 7.51% crude fiber, 2.12% calcium, 0.58% phosphorus, 18.87% ash, 19.60% crude protein, 1.95% non-protein nitrogen, 9.79% acid detergent fiber (ADF), 21.63% neutral detergent fiber (NDF), 2.87% ether extract (acid hydrolysis), and 10.04% moisture (volatile at 105 °C).

The same protein-energy supplement served as the base formulation for the experimental group, which received an additional 1.5% postbiotic derived from *Saccharomyces cerevisiae*, corresponding to 15 g of postbiotic per kg of supplement. This formulation resulted in an average daily intake of 10.5 g of postbiotic per animal. The *Saccharomyces cerevisiae* yeast culture used as a postbiotic consisted of a standardized mixture of inactivated microbial biomass and metabolites generated during a controlled fermentation process. Its composition included cellular fractions and a set of bioactive compounds released into the fermentation medium, including peptides, alcohols, esters, oligosaccharides, organic acids, B-complex vitamins, and nucleotides (16, 22). The detailed product specification, including manufacturing and standardization parameters, were provided by the manufacturer (Aleris – Comércio e Exportação de Produtos para Nutrição Ltda.).

The mineral supplement was formulated with the following guaranteed levels: calcium 160–200 g/kg (min/max), phosphorus 40 g/kg (min), cobalt 60 mg/kg (min), copper 750 mg/kg (min), sulfur 10 g/kg (min), fluoride 400 mg/kg (max), iodine 60 mg/kg (min), magnesium 5,000 mg/kg (min), manganese 825 mg/kg (min), selenium 15 mg/kg (min), sodium 150 g/kg (min), and zinc 2,700 mg/kg (min). The experimental groups were managed in separate paddocks, and only animals from the same group had *ad libitum* access to the same feed bunk. Thus, although paddock rotation was carried out during management, no cross-contamination between diets occurred.

### 2.4 Oral clinical examination

The oral clinical status of the 150 heifers was established through intraoral examinations and periodontal assessments of all eight incisor teeth, conducted monthly. A total of four evaluations were performed throughout the experiment, resulting in the assessment of 4.800 deciduous teeth. The indicators used to characterize the lesions were based on the visual appearance of the teeth, combined with gingival margin probing using a millimeter-marked Williams periodontal probe. The probe was gently inserted into the gingival sulcus and positioned parallel to the gingival margin on the labial surface, moving uniformly along the margin of each incisor (1). All clinical examinations were performed by a single evaluator who had been previously trained to standardize the diagnostic criteria for periodontal disease. The information obtained was recorded on a specific form (odontogram), ensuring uniformity in data collection.

Gingivitis was defined by the presence of inflammatory changes in the gingival borders, such as edema, color alteration, and spontaneous or probe-induced bleeding. Periodontal sites were considered healthy if they exhibited no signs of inflammation in the gingival margins and no other clinical indications of periodontal alterations.

### 2.5 Statistical analysis

A Repeated Measures ANOVA (RM-ANOVA) was employed to examine statistically significant differences (*p* < 0.05) within-group (protein-energetic plus postbiotic supplement group, protein-energetic supplement group, and mineral supplement group) in the variables “weight gain” and “cases of gingivitis.” To verify the assumptions of the analysis, tests for residual normality (Shapiro–Wilk), sphericity (Mauchly’s test), and homogeneity of variances (Levene’s test) were conducted. When assumptions were violated, the Friedman ANOVA, a non-parametric alternative, was applied. For significant results, effect sizes were calculated using Cohen’s d for RM-ANOVA and Wilcoxon’s r for Friedman ANOVA. *Post hoc* analyses were conducted using the Bonferroni test (RM-ANOVA) and the Wilcoxon Signed-Rank test (Friedman ANOVA) to identify specific within-group differences.

A one-way ANOVA was performed to evaluate statistically significant differences (*p* < 0.05) between groups for the variables “weight gain” and “cases of gingivitis.” The normality of distributions was assessed using the Shapiro–Wilk test, and homogeneity of variances was tested using Levene’s test. In cases where assumptions were not met, the Kruskal–Wallis test was used as a non-parametric alternative. For significant outcomes, effect sizes were measured using Cohen’s d (for one-way ANOVA) and the r coefficient (for Kruskal–Wallis). For multiple comparisons, the Bonferroni test was applied following one-way ANOVA, and the Dunn test was used after the Kruskal–Wallis test to identify specific between-group differences. All graphical outputs were performed using R software (version 4.4.2), with scripts developed specifically for this study (23). The figures were generated directly in R Studio using appropriate packages for data visualization and graphical analysis.

## 3 Results

**Figure 1:**
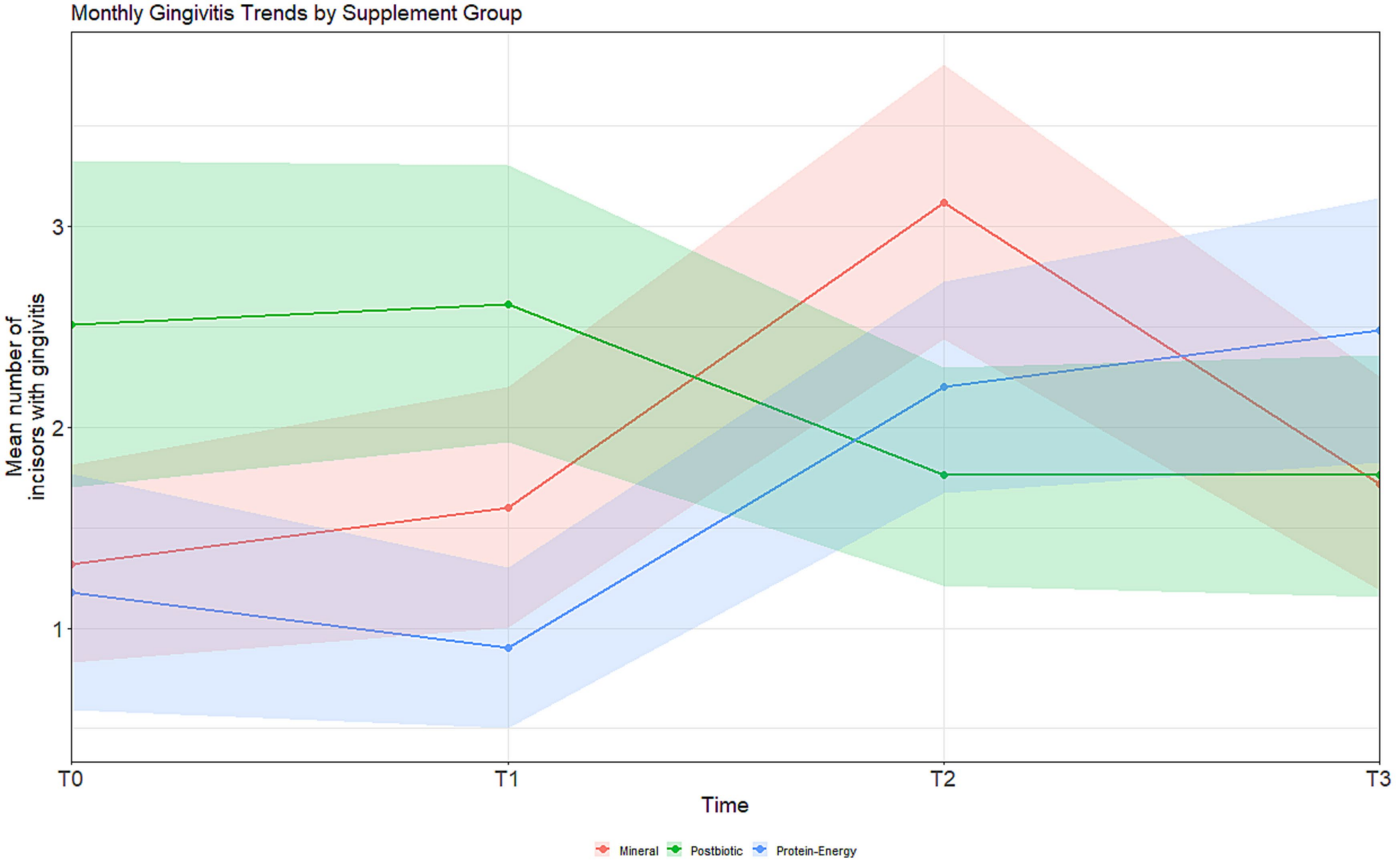
Monthly trends in the mean number of incisors with gingivitis in Brangus heifers receiving mineral supplement, protein–energy supplement, or postbiotic protein–energy supplement over a 4-month period. Shaded areas represent the 95% confidence interval.

The results of the descriptive statistical analysis of the evaluated groups are presented in Tables 1, 2. Considering the intra-group analyses for the variable gingivitis, no statistically significant differences were found in the group that received the protein-energy supplement with *S. cerevisiae* postbiotic (*p* < 0.195). In the protein-energy supplement group, a significant increase in the number of gingivitis cases was observed over time [χ^2^(3) = 35.573, *p* < 0.001; Kendall’s *W* = 0.237], with *post-hoc* test results and effect sizes summarized in Table 3. Similarly, in the mineral supplement group, a significant intra-group increase in gingivitis cases was also detected [*F*(3) = 7.265, *p* < 0.001; partial η^2^ = 0.129], with detailed results provided in Table 4. The between-group analysis for the variable gingivitis, both at baseline (T0) and at the end of the experiment (T3), revealed no statistically significant differences (Table 5). These temporal patterns are illustrated in Figure 1.

**Table 1:** Descriptive data of average body weight (kg) for the postbiotic protein-energy supplement group (*n* = 50), the standard protein-energy supplement group (*n* = 50), and the mineral supplement group (*n* = 50), evaluated monthly during the present study.

Group	Moment	Mean weight	95% CI mean		Std. deviation	95% CI Std. Dev		Coefficient of variation
			Upper	Lower		Upper	Lower	
Postbiotic protein-energy supplement	T0	250.72	258.446	243.003	26.881	30.904	21.919	0.107
	T1	261.91	270.729	253.087	30.71	35.948	24.667	0.117
	T2	264.68	273.722	255.646	31.466	37.56	24.648	0.119
	T3	282.19	291.934	272.454	33.908	42.349	25.419	0.12
Protein-energy supplement	T0	250.31	257.904	242.716	26.721	30.821	21.833	0.107
	T1	261.12	268.837	253.403	27.155	31.018	22.516	0.104
	T2	273.76	281.969	265.551	28.884	32.712	24.17	0.106
	T3	277.38	286.277	268.483	31.304	36.134	25.637	0.113
Mineral supplement	T0	251.28	260.17	242.39	31.28	37.029	25.156	0.124
	T1	257.58	266.138	249.022	30.113	35.594	23.725	0.117
	T2	259.08	267.501	250.659	29.632	35.037	23.018	0.114
	T3	263.54	271.621	255.459	28.434	34.38	21.824	0.108

**Table 2:** Descriptive data of the mean number of deciduous incisors with gingivitis in the postbiotic protein-energy supplement group (*n* = 50), the standard protein-energy supplement group (*n* = 50), and the mineral supplement group (*n* = 50), evaluated monthly during the present study.

Group	Moment	Mean	95% CI Mean		Std. deviation	95% CI Std. Dev.		Coefficient of variation
			Upper	Lower		Upper	Lower	
Postbiotic protein-energy supplement	T0	2.51	3.323	1.698	2.829	3.213	2.226	1.127
	T1	2.612	3.301	1.924	2.396	2.699	1.967	0.917
	T2	1.755	2.297	1.213	1.888	2.202	1.491	1.076
	T3	1.755	2.357	1.153	2.097	2.477	1.648	1.195
Protein-energy supplement	T0	1.18	1.77	0.59	2.077	2.581	1.319	1.76
	T1	0.9	1.299	0.501	1.403	1.928	0.908	1.559
	T2	2.2	2.726	1.674	1.852	2.17	1.463	0.842
	T3	2.48	3.138	1.822	2.314	2.655	1.834	0.933
Mineral supplement	T0	1.32	1.812	0.828	1.731	2.04	1.289	1.312
	T1	1.6	2.199	1.001	2.109	2.443	1.636	1.318
	T2	3.12	3.801	2.439	2.396	2.709	1.99	0.768
	T3	1.72	2.249	1.191	1.863	2.195	1.472	1.083

**Table 3:** Intra-group comparison for the gingivitis variable in the standard protein-energy supplement group (*n* = 50) across the four time points evaluated in the present study.

Comparison 1	Comparison 2	*T*-Stat	df	*W*_i_	*W*_j_	*r*_rb_	*p*	*p*_bonf_	*p*_holm_
T0	T1	0.396	147	103.000	99.000	0.135	0.693	1.000	1.000
	T2	4.258	147	103.000	146.000	−0.480	<0.001	<0.001	<0.001
	T3	4.852	147	103.000	152.000	−0.614	<0.001	<0.001	<0.001
T1	T2	4.654	147	99.000	146.000	−0.755	<0.001	<0.001	<0.001
	T3	5.248	147	99.000	152.000	−0.750	<0.001	<0.001	<0.001
T2	T3	0.594	147	146.000	152.000	−0.082	0.553	1.000	1.000

Grouped by subject. Rank-biserial correlation based on individual signed-rank tests.

**Table 4:** Intra-group comparison for the gingivitis variable in the mineral supplement group (*n* = 50) across the four time points evaluated in the present study.

Comparison 1	Comparison 2	Mean difference	95% CI Mean		SE	*t*	Cohen’s *d*	95% for Cohen’s *d*		*p*_bonf_
			Lower	Upper				Lower	Upper	
T0	T1	−0.280	−1.399	0.839	0.407	−0.688	−0.137	−0.687	0.413	1.000
	T2	−1.800	−3.015	−0.585	0.442	−4.075	−0.882	−1.526	−0.238	0.001**
	T3	−0.400	−1.316	0.516	0.333	−1.200	−0.196	−0.648	0.256	1.000
T1	T2	−1.520	−2.755	−0.285	0.449	−3.383	−0.745	−1.385	−0.105	0.008**
	T3	−0.120	−1.282	1.042	0.422	−0.284	−0.059	−0.628	0.511	1.000
T2	T3	1.400	0.121	2.679	0.465	3,010	0.686	0.031	1.341	0.025*

p-value and confidence intervals adjusted for comparing a family of 6 estimates (confidence intervals corrected using the Bonferroni method). *p < 0.05, **p < 0.01.

**Table 5:** Comparative analysis of the gingivitis variable among the groups used in the present study.

Comparison	*z*	*W*_i_	*W*_j_	*r*_rb_	*p*	*p*_bonf_	*p*_holm_
Postbiotic protein-energy supplement *vs.* protein-energy supplement	−1.806	69.265	84.51	0.2	0.071	0.213	0.213
Postbiotic protein-energy supplement *vs* Mineral supplement	−0.219	69.265	71.11	0.029	0.827	1	0.827
Protein-energy supplement *vs* mineral supplement	1.596	84.51	71.11	0.184	0.111	0.332	0.221

**Figure 2:**
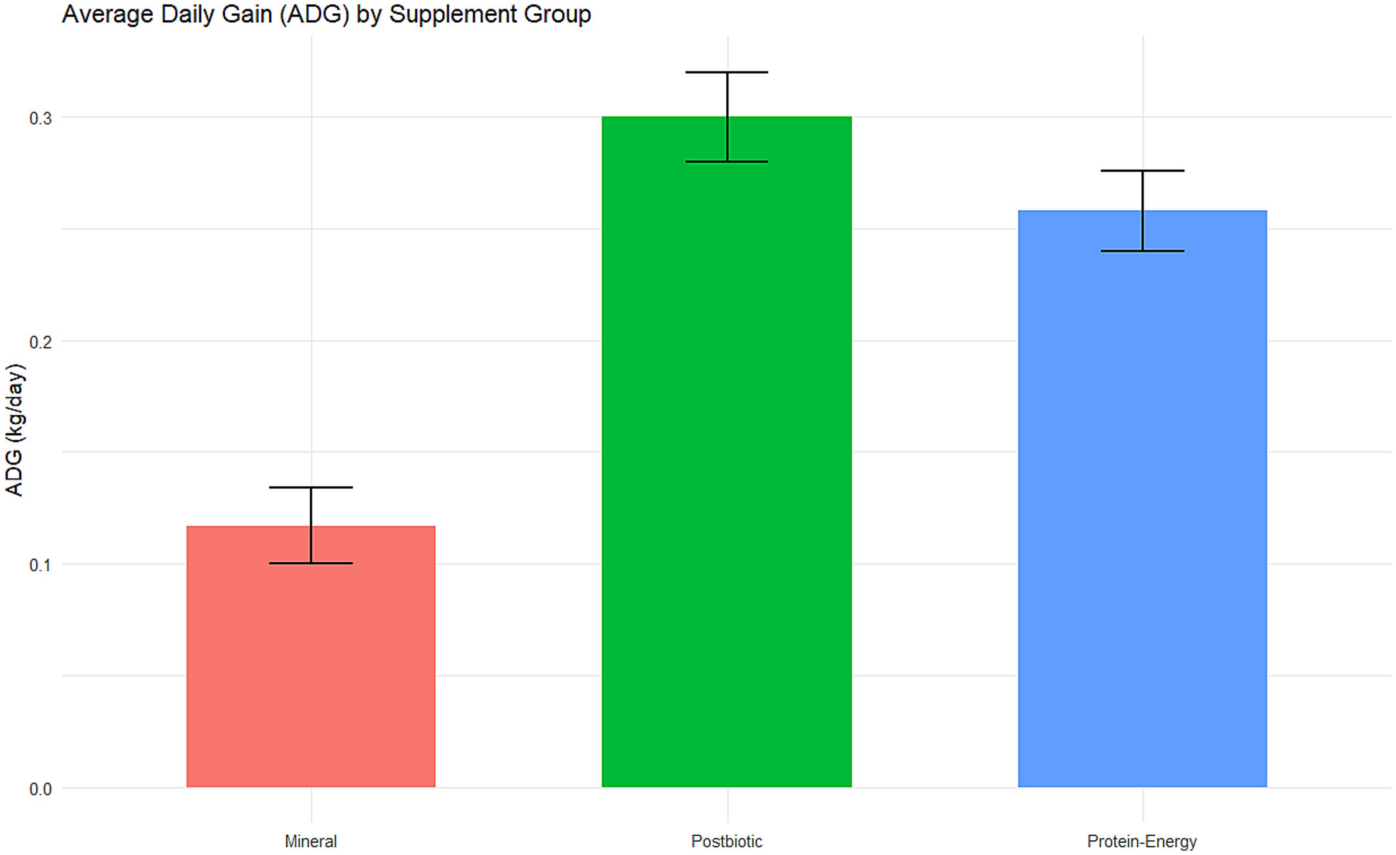
Average daily gain (ADG) of heifers receiving mineral supplement, protein–energy supplement, or postbiotic protein–energy supplement over a 4-month period. Bars represent mean values, and error bars indicate the 95% confidence interval.

Regarding the weight variable, the average daily gain (ADG) at the end of the experiment was 0.300 kg/day in the group that received the protein-energy supplement with postbiotic, 0.258 kg/day in the protein-energy supplement group, and 0.117 kg/day in the mineral supplement group. Between-group analyses for weight showed that animals in the postbiotic group had significantly higher weight gain compared to those in the standard protein-energy group (df = 2; *F* = 4.76; *p* = 0.011; Cohen’s *d* = 0.596). These differences in ADG among dietary treatments are illustrated in Figure 2.

## 4 Discussion

The use of a protein-energy supplement containing *Saccharomyces cerevisiae* postbiotic did not reduce the frequency of gingivitis episodes in the deciduous dentition of the heifers. On the other hand, intra-group analysis also did not show a significant increase in the number of teeth affected by gingivitis in these animals. In contrast, the present study revealed that the group receiving the standard protein-energy supplement exhibited an increase in the number of teeth with gingivitis over time, as indicated by the intra-group analyses and Figure 1. These results are consistent with the findings of Saraiva et al. (6), who reported a similar trend in young cattle fed with protein supplements.

Unlike classical infectious diseases, periodontal diseases may be associated with environmental and genetic factors that influence susceptibility to their development and the progression of their various clinical manifestations (24). Moreover, the individual variability observed in cattle maintained under the same feeding regime (6) may help explain the intra-group differences identified over time, both in the group that received the protein-energy supplement and in the group that received the mineral supplement.

Early loss of incisor teeth, whether deciduous or permanent, can impair feeding efficiency and negatively affect the productive performance of animals (9). In the present study, the heifers evaluated are future breeding stock within the production system, so partial assessment of the incisor teeth represents a relevant indicator of the severity of these disorders within the herd. Thus, the group that received the postbiotic showed an improvement in average daily weight gain and stability in the occurrence of gingivitis episodes in the deciduous incisor teeth (Figure 2). Although the direct economic impacts were not assessed, preventing more severe forms of periodontal diseases, represents an important preventive strategy for cattle maintained in pasture-based systems.

In humans, studies have shown that the diversity of dietary nutrients can influence periodontal health through modulation of the oral microbiome (25–27). Although recent research has advanced in characterizing the oral microbiota of cattle with periodontal diseases (28–30), information remains scarce regarding the effects of dietary supplements and additives on the composition of the bovine oral microbiota.

The microbiological characterization of the oral cavity provides valuable insights into microbial dynamics. In the present study, this analysis was not included due to methodological limitations; however, it is planned for future stages of the research. It is known that a reduction in the frequency and duration of gingivitis episodes can be used as an indirect criterion to measure biological events related to the progression to periodontitis, the most severe and irreversible form of periodontal disease in cattle (9). In this context, the present study contributes by providing a general characterization of the epidemiological aspects associated with the occurrence of gingivitis in female heifers during the rearing phase, as well as suggesting possible intervention strategies for the control of these diseases.

In cattle, the use of virginiamycin has shown preventive and control effects, reducing the occurrence of gingivitis and necrotizing gingivitis (31, 32). Similar results have been observed in beef cattle with aggressive periodontitis, where virginiamycin use led to a reduction in the prevalence of oral diseases and improvements in clinical conditions and overall animal health (3, 33). However, given the current restrictions on antibiotic use in animal production, there is growing interest in safe and sustainable alternatives, such as probiotics, prebiotics, and postbiotics (11, 12).

In this context, the use of probiotics and postbiotics derived from *S. cerevisiae* has gained prominence, offering various benefits to cattle health and performance, including improved feed efficiency and ruminal fermentation, modulation of the immune response, reduced feed intake, and prevention of gastrointestinal disorders such as acidosis (34–37). However, the multifactorial nature of periodontal diseases poses methodological challenges to their investigation, making it difficult to identify in isolation all the factors involved in the etiopathogenesis of these conditions, such as metabolic and genetic aspects and pasture variability, which underscores the complexity of studying oral health in cattle and other ruminants.

Although the animals in the group receiving the protein-energy supplement with postbiotic showed greater weight gain at the end of the experimental period, no clear therapeutic effects on the oral health of heifers in the rearing phase were observed. Nevertheless, it is plausible not to dismiss a possible modulatory role of the postbiotic on the animals’ oral microbiota, especially since, among the tested groups, only this group did not show an increase in the number of gingivitis cases in the intra-group analysis.

The animals that received the mineral supplement showed an increase in the occurrence of intra-group gingivitis episodes over time; however, this increase was not sufficient to progress to cases of periodontitis, nor did it result in statistically significant differences compared to the other tested groups. It is likely that the animals’ natural immune response and possible external factors contributed to containing the progression of lesions in these animals.

The findings of this study indicate that postbiotic supplementation did not significantly alter the overall occurrence of gingivitis but contributed to maintaining stable gingival conditions and improving growth performance in grazing heifers. These results suggest that nutritional modulation may influence early inflammatory processes in the oral cavity, even in the absence of detectable clinical differences among groups. Although zootechnical benefits have been observed in animals supplemented with *S. cerevisiae*-derived postbiotics, further research in broader production systems is needed to investigate the mechanisms influencing oral health in ruminants.

Integrating microbiome, metabolomic, and immunological analyses will clarify whether postbiotics modulate local inflammatory responses or microbiota composition within the oral cavity. Understanding these mechanisms could lead to the development of targeted nutritional strategies for the prevention of periodontal diseases and other inflammatory conditions in ruminants. Moreover, it is possible that exploring the oral–ruminal axis may reveal new insights into how nutritional additives contribute to both oral homeostasis and overall productivity in sustainable grazing systems. This becomes relevant considering that dysbiotic communities and an inflammatory environment in the dental biofilm and rumen of cattle with periodontitis have already been previously described (29).

## 5 Conclusion

The results of the present study suggest that *S. cerevisiae* postbiotic, when combined with protein supplementation, may contribute to the maintenance of gingival health and improved zootechnical performance in heifers, even without a direct therapeutic effect on gingivitis. This represents a promising nutritional strategy, whose mechanism of action still needs to be elucidated.

## Data Availability

The raw data supporting the conclusions of this article will be made available by the authors, without undue reservation.
